# Transcription of the rat testis-specific *Rtdpoz-T1 *and *-T2 *retrogenes during embryo development: co-transcription and frequent exonisation of transposable element sequences

**DOI:** 10.1186/1471-2199-10-74

**Published:** 2009-07-25

**Authors:** Chiu-Jung Huang, Wan-Yi Lin, Che-Ming Chang, Kong-Bung Choo

**Affiliations:** 1 Department of Animal Science, School of Agriculture, Chinese Culture University, Yang-Ming-Shan, Taipei, Taiwan; 2 Graduate Institute of Biotechnology, School of Agriculture, Chinese Culture University, Yang-Ming-Shan, Taipei, Taiwan; 3 Department of Medical Research and Education, Taipei Veterans General Hospital, Shipai, Taipei, Taiwan; 4 Graduate Program, Department of Biotechnology and Laboratory Science in Medicine, School of Biomedical Science and Engineering, National Yang Ming University, Shipai, Taipei, Taiwan; 5 Institute of Clinical Medicine, School of Medicine, National Yang Ming University, Shipai, Taipei, Taiwan

## Abstract

**Background:**

Retrotransposition is an important evolutionary force for the creation of new and potentially functional intronless genes which are collectively called retrogenes. Many retrogenes are expressed in the testis and the gene products have been shown to actively participate in spermatogenesis and other unique functions of the male germline. We have previously reported a cluster of retrogenes in the rat genome that encode putative TRAF- and POZ-domain proteins. Two of the genes, *Rtdpoz-T1 and -T2 *(abbreviated as *T1 *and *T2*), have further been shown to be expressed specifically in the rat testis.

**Results:**

We show here that the *T1 *and *T2 *genes are also expressed in the rat embryo up to days 16–17 of development when the genes are silenced until being re-activated in the adult testis. On database interrogation, we find that some *T1/T2 *exons are chromosomally duplicated as cassettes of 2 or 3 exons consistent with retro-duplication. The embryonic *T1/T2 *transcripts, characterised by RT-PCR-cloning and rapid amplification of cDNA ends, are further found to have acquired one or more noncoding exons in the 5'-untranslated region (5'-UTR). Most importantly, the *T1*/*T2 *locus is embedded within a dense field of relics of transposable element (TE) derived mainly from LINE1 and ERV sequences, and the TE sequences are frequently exonised through alternative splicing to form the 5'-UTR sequences of the *T1/T2 *transcripts. In a case of *T1 *transcript, the 3'-end is extended into and terminated within an L1 sequence. Since the two genes share a common exon 1 and are, therefore, regulated by a single promoter, a *T2*-to-*T1 *co-transcription model is proposed. We further demonstrate that the exonised 5'-UTR TE sequences could lead to the creation of upstream open reading frames resulting in translational repression.

**Conclusion:**

Exonisation of TE sequences is a frequent event in the transcription of retrogenes during embryonic development and in the testis and may contribute to post-transcriptional regulation of expression of retrogenes.

## Background

Retrotransposition is an important evolutionary driving force for the creation of new genes with novel lineage- and species-specific phenotypic traits. New genes created through retrotransposition are retrogenes that are devoid of introns. Furthermore, paralogues are subsequently created through segmental duplications and sequence modifications. Retrogenes could be re-activated by putative promoters and other transcription regulatory elements suitably located upstream of the retrogene insertion sites [1-3]. In the process of transcriptional re-activation, the newly arisen transcript may acquire one or more noncoding exons in the 5'-untranslated region (5'-UTR). In the context of our current knowledge of the generation of multiple transcripts from a single gene through alternative splicing [4,5], the term "retrogene" is used throughout this paper to mean the "genomic copy" of a gene that is consituted of a complete coding sequence without intron interruption taking no consideration on whether the resulting transcripts carry exonised sequences through alternative splicing. Whether or not "retrogene" should be redefined as such is debatable.

Many retrogenes are functional [6]. It has further been estimated that there are in excess of 1,000 transcribed retrocopies in the human genome over a tenth of which is biologically active [7]. Interestingly, the bulk of retrogenes is preferably expressed in the testis where the retrogene products actively participate in the spermatogenesis process and serve to further enhance biological functions unique to the male germline [8,9]. Transcription in the testis is not as tightly regulated as in other somatic tissues due to hyper transcription rates which could result in non-discriminatory activation of otherwise imperfect or weak promoters [10]. Such a mode of promiscuous transcription, and possibly erratic alternative splicing processes, could lead to the generation of fortuitous testicular transcripts. Promiscuous transcription and transient transcriptional gene activation have also been shown to occur at the crucial stage of zygotic genome activation [11]. One outstanding feature of promiscuos transcription is excessive transcription of highly repetitive genomic sequences [11,12]. The bulk of genomic repetitive sequences are transposable elements (TEs). A significant number of mammalian genes has been shown to be regulated by transcriptional elements of the endogenous retroviruses (ERVs) or long-terminal repeats (LTRs) of TEs [13]. Evidence is emerging to suggest that ERVs and other TEs may constitute a critical driving force in speciation [14].

We have previously proposed the existence of a novel bipartite TDPOZ protein family members of which carry the TD (TRAF domain), also called MATH (meprin and TRAF homology, and POZ (poxvirus and zinc finger)/BTB (Broad complex, Tramtrack, Bric à brac) [15]. Almost all known eukaryotic TD proteins are known to be involved in the regulation of protein processing and ubiquitination [16]. The representative TD proteins, the tumour necrosis factor receptor-associated factors (TRAFs), bind to the tumour necrosis factor receptors or other adaptor molecules to participate in cellular proliferation and survival, and in cell-death signalling [17,18]. On the other hand, POZ proteins have been implicated in biological processes including DNA damage responses, cell cycle progression and in embryonic developmental events and hematopoietic stem cell fate determination [19]. The TD and POZ domains are found in separate proteins in association with other DNA-binding or protein-protein interacting domains except for the TDPOZ bipartite proteins that we have first reported [15,20]. *Tdpoz *genes are found in both higher and lower animals and in plants suggesting important biological functions. To date, the only functionally characterised mammalian TDPOZ protein is the nuclear speckle-associated protein SPOP. SPOP acts as an adaptor of Daxx in the ubiquitination process involving CUL3-based ubiquitin ligase contributing to regulation of Hedgehog/Gli signalling [21-24].

**Figure 1:**
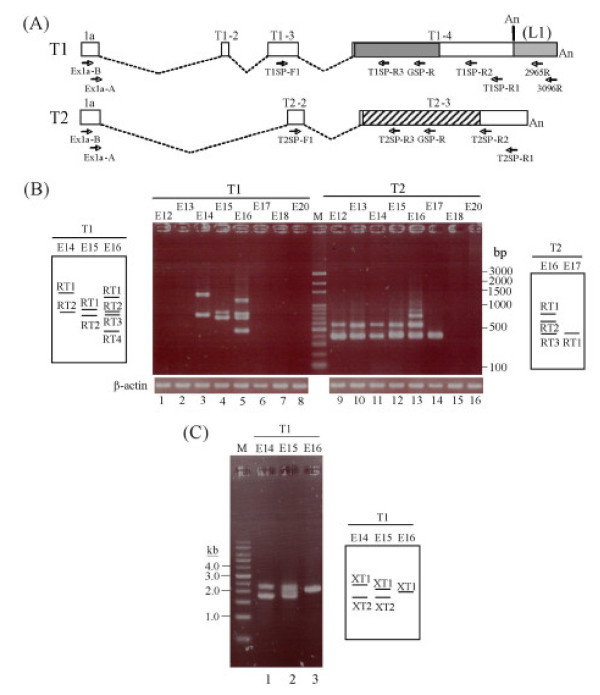
**Developmental regulation of *Rtdpoz-T1 *and *-T2***. **(A) **Exons constituting the testicular *T1 *and *T2 *transcripts. In both *T1 *and *T2*, the common exon 1a is used. The noncoding sequences are shown as unfilled boxes. The uninterrupted *T1*- and *T2-*coding sequences that reside in exons 4 (T1–4) and 3 (T2–3) of the respective genes are shown as cross-hatched or slanting-hatched boxes, respectively. In *T1*, the L1 sequence (see text) in the 3'-UTR is shown in grey. The relative positions of the primers used in the RT-PCR expression profiling and the RACE analysis are shown (see Table 2 for primer sequences). An, polyA tract. **(B) **Developmental expression profiles of the *T1 *and *T2 *genes. The developmental stages analysed were from day 12 (E12) through to day 20 (E20). β-actin was included as a control. In the experiments, the Ex1a-B + T1SP-R1 or Ex1a-B + T2SP-R1 primer pairs (see above) were used in the first-round PCR for *T1 *and *T2*, respectively, followed by the use of the Ex1a-A + T1SP-R3 (for *T1*) or Ex1a-A + T2SP-R3 (for *T2*) primer pairs in the second-round PCR as detailed in the Methods section. On the left and right of the photo panels are schematic representations of the PCR bands with band designations as explained in the text. **(C) **3'-Extended RT-PCR analysis of the *T1 *transcripts in the developmental stages that expressed the gene. In the first-round PCR, the Ex1a-B and 3096R primers (see **(A) **above) were used followed by a second-round PCR using the Ex1a-A and 2965R primers as described in Methods. Band designations, prefixed by "XT", are depicted in the schematic drawing alongside the gel display.

In the mouse and rat genomes, *Tdpoz *sequences appear in multiplicity as retrocopies of full-length or truncated coding sequences uninterrupted by introns [15,20,25]. *Tdpoz *retrogene multiplicity is found only in the two completely sequenced rodent genomes and not in other animals and plants suggesting that the *Tdpoz *retrogenes emerged after the evolutionary divergence of the rodent lineage. The first reported mouse *Tdpoz *retrogene, *Tdpoz1 *[GenBank:AF290198], is transcribed in the egg and in the pre-implantation-stage embryo [15,20]. Other mouse *Tdpoz *retrogenes are subsequently identified and are found to be transcribed in low levels in the pre-implantation embryo and in the testis [15]. In the rat, the *Rtdpoz-T1 *and -*T2 *genes (abbreviated as *T1 *and *T2 *herein) [GenBank:AY902365 and AY902367, respectively] are transcribed specifically in the testis [20]. Database interrogations of the rat genome have further revealed ~300 hits of *Tdpoz *homologous sequences, dubbed *Rtdpoz *for rat *Tdpoz*, that show >85% sequence identities with the *T1 *or *T2 *open reading frame (ORF); these sequence hits are distributed over seven different chromosomes of the rat genome. However, the bulk of the hits is found in a 2.5-Mb cluster in the Rn2_2148 supercontig [GenBank:NW_047626.2] mapping at 2q34 on chromosome 2 [20]. Active retrotransposition and duplication are thought to be the major forces driving the creation and expansion of the *Rtdpoz *sequence repertoire in the contemporary rat genome. In a ~800-kb region at the 2q34 locus, twenty-six *Rtdpoz *retrogenes are discerned, including *T1 *and *T2 *[20]. The biological functions of the putative T1 and T2 proteins have yet to be elucidated. On alignment of the cDNA and genomic DNA sequences, it has been determined that the major 3'-terminal exon of the testicular *T1 *and *T2 *transcripts carry the uninterrupted coding sequences, qualifying *T1 *and *T2 *as retrogenes; however, the transcripts have also acquired two to three short noncoding exons in the 5'-untranslated region (5'-UTR) of the transcripts (Figure 1A). Intriguingly, all the *T1 *and *T2 *transcripts share a common 5'-leader exon 1a sequence that appears only once at 2q34 and is also unique in the entire rat genome. How exon 1a is added to the 5'-ends of both the *T1 *and *T2 *transcripts remains to be addressed. We have also previously reported a minor testicular transcript, dubbed *T3*, which appears to be a fusion of *T1 *and *T2 *exons [20]. Are there other *T1-T2 *"chimeric" transcripts, and how are these transcripts generated? In this work, we further explore other novel features in the *T1 *and *T2 *transcripts in an attempt to further understand transcription and pre-mRNA processing of retrogenes.

## Results

### Developmental regulation of *T1 *and *T2 *expression

To determine the expression status of the *T1 *and *T2 *genes during development, RNA was prepared from developmental stages between day 12 (E12) to day 20 (E20) just before birth. RT-PCR was performed using *T1*- or *T2*-discriminating primers located in different exon sequences of the genes (Figure 1A). The RT-PCR results show that *T1 *was expressed only at stages E14 to E16 of development (Figure 1B, lanes 3–5) with a distinctive expression profile for each stage indicating differential *T1 *transcription at these developmental stages. On the other hand, *T2 *was expressed up to E17 and the expression profile was rather consistent and was largely similar to that of the testis [20] except that at E16, extra bands were detected, and at E17 only the lower band was present (Figure 1B, lanes 9–14). The transcription profiling experiments, hence, establish that expression of the testicular *T1 *and *T2 *genes is developmentally regulated, and that there exists a notable disparity in the expression patterns of the genes at specific developmental stages. This suggests that transcription of the two genes, despite the sharing of the leader exon 1a, is differentially regulated. The *T1 *and *T2 *genes, are silenced at day 17 (E17) and day 18 (E18) of development, respectively, when organogenesis is now completed and the foetus enters the active phase of growth and expansion.

### Assorted 5'-UTR structures derived from alternative splicing involving transposable element sequences

**Figure 2:**
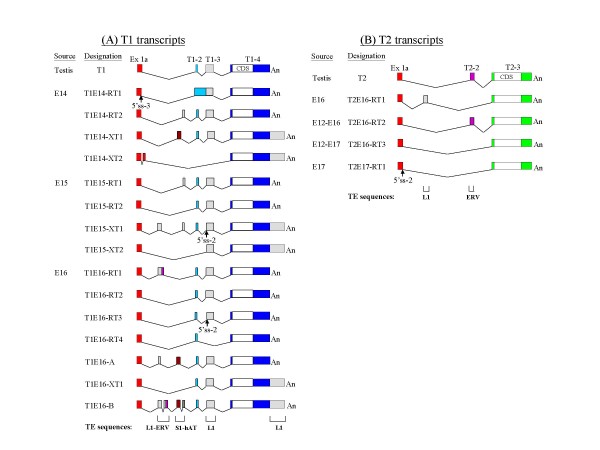
**Exon map of the embryonic *T1 *(A) and *T2 *(B) transcripts**. The *T1 *and *T2 *transcripts shown were derived by a combination of direct standard RT-PCR (transcripts designations with the "RT" prefix), 3'-extended RT-PCR (XT-tagged transcripts) and RACE experiments (transcripts T1E16-A and -B) as described in the text. For identical *T1 *or *T2 *transcripts derived from the various experimental approaches, only a representative transcript designation is used. Included as a reference is the testicular *T1 *and *T2 *exon map that was previously derived [20]. Exons are represented by coloured blocks and the exon designations are shown above the testicular transcripts. The coding sequences (CDS) of *T1 *and *T2 *are shown as white boxes. An, polyA tract. Alternative 5'-splice sites (5'ss) are indicated. At the bottom of the *T1 *and *T2 *transcript panels, the elucidated transposable element sequences are displayed aligning with the corresponding exon of the transcripts. L1, LINE1 sequence; ERV, endogenous viral sequence; S1, SINE1 sequence; hAT, DNA transposon. T1E16-A and -B are now filed as GenBank:FJ004892 and GenBank:FJ004893, respectively. The scheme is not drawn to scale.

To fully characterise the multiple *T1 *and *T2 *transcripts, a combination of RT-PCR and rapid amplification of cDNA ends (RACE) approaches was applied. In the RT-PCR approach, each of the RT-PCR bands was cloned and two to three clones generated from each RT-PCR product were randomly selected for sequence analysis (Figure 1B). In this effort, a total of eleven unique *T1 *and *T2 *transcript sequences were derived for all the expressing stages (Figure 2A and see below; designations of the RT-PCR-derived transcripts carry the prefix "RT" in Figure 1B in the schematic drawings in the left and right panels). To define the 5'- and 3'-ends, RACE experiments were conducted. To avoid discrimination between *T1 *and *T2 *in the RACE analysis and to increase specificity and sensitivity in detecting transcripts of low abundance, *T1 *and *T2 *consensus primers were used in the first-round cDNA synthesis followed by second-round nested PCR using *T1*- or *T2*-specific primers. A total of forty-nine 5'-RACE and twenty-five 3'-RACE clones were obtained and sequenced. The vast majority of the 5'RACE sequences were identical with the *T1 *and *T2 *transcript sequences obtained in the RT-PCR experiments above. Moreover, all *T1 *and *T2 *transcripts carried the leader exon 1a and the 5'-termini were largely similar to those first reported for the testicular transcripts [20]. In the 3'-RACE analysis, the sequences obtained for the *T2 *transcripts at all developmental stages were identical to the 3'-end of the testicular *T2 *gene and were used to construct the full *T2 *transcript sequences (Figure 2B). For the *T1 *transcripts, all except one 3'-RACE sequences were identical to the 3'-end of the testicular *T1 *transcript. The novel *T1 *3'-terminal sequence (contained in the *T1 *transcripts designated T1E16-B in Figure 2A) was a 542-bp 3'-extension of the regular 3'-end for the *T1 *transcripts; the extended 3' sequence also possesses a putative but non-canonical ATAAAA polyadenylation signal located 6-bp upstream of the polyA tail (see GenBank:FJ004893 for sequence details). A query of the GIRI RepBase database further revealed that the extension was a segment of the non-long terminal repeat (non-LTR) long interspersed element 1 (LINE1, or L1) sequence (Figure 2A). On the other hand, a similar but shorter transcript, T1E16-A [GenBank:FJ004892], lacks this L1 extension (Figure 2A).

The uncovering in the 3'-RACE experiments of the extended 3'-UTR sequence of *T1 *warranted further RT-PCR analysis to confirm the authenticity of the 3'-extension and to further investigate if other *T1 *transcripts carried similar 3'-extension. To achieve this goal, two-round nested RT-PCR was performed across the entire gene sequences stretching from the 5'-terminal exon 1a to the extended L1 sequence using primers Ex1a-B and 3096R followed by the use of primers Ex1a-A and 2965R (Figure 1A, T1 primer map). In the three *T1*-expressing developmental stages, one or two RT-PCR bands were discerned indicating, indeed, the existence of multiple *T1 *transcripts with the 3'-extension (Figure 1C). The PCR products were subsequently cloned and sequenced and the sequences are designated with the prefix "XT" (Figure 2A). Full-length XT transcripts are constructed assuming they share the same testicular 5'- and the 3'-end of T1E16-B (Figure 2A). A notable exception in the XT transcripts was T1E14-XT2 which included a new 45-bp exon the sequence of which was located 887-bp downstream of the leader exon 1a; this transcript also lacks the ubiquitous exons 2 and 3 (T1–2 and -3) of *T1 *(Figure 2A).

**Figure 3:**
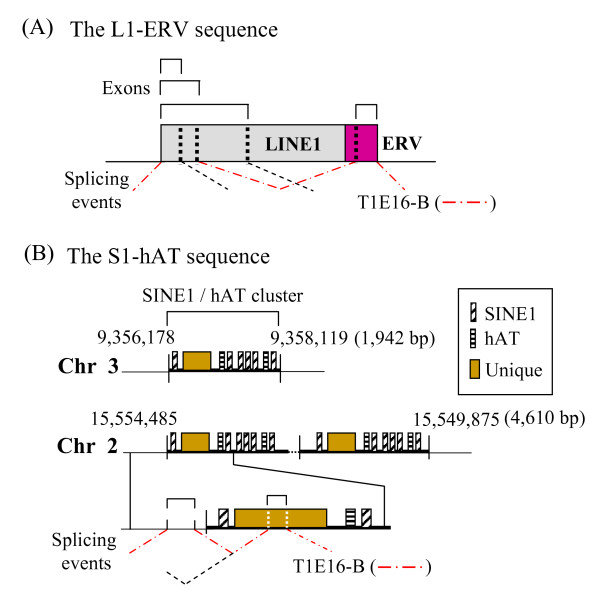
**Alternative splicing of two genomic regions of transposable elements with contribution to *T1 *transcripts**. The genomic regions are as defined in Figure 2A and Table 1. Sequence details are found in Additional file 1. **(A) **The L1-ERV segment is a composite of LINE1 (L1) (in grey) and ERV (in purple) sequences. The roofs above the TE sequences represent exons resulting from alternative splicing as demarcated by vertical dotted lines in the boxes representing TEs. Slanting dashed lines in black denote splicing events that generate the discerned exons. The dashed-dotted lines in red indicate two consecutive splicing events in this region that have led to the two TE-associated exons in the T1E16-B transcript. **(B) **The SINE1 (S1)-hAT sequence as defined in the text. There are two tandem copies of S1-hAT on chromosome 2 (in the Rn2_2148 supercontig [GenBank:NW_047626.2], RGSC ver3.4) and a single copy on chromosome 3 (Rn3_2179 supercontig, [GenBank:NW_047657.2]) of the rat genome; the nucleotide positions and the sizes of the respective sequence copies in these chromosomal segments are indicated. At the bottom, splicing events and the resulting exons are depicted using the same symbols as for **(A) **above. The scheme is not drawn to scale.

The sequences generated by RT-PCR and RACE were combined to construct full-length *T1 *and *T2 *transcript sequences as follows. For the *T1 *transcripts, 3'-RACE experiments generated two authentic 3'-ends: the first is as found in the testicular *T1 *transcript and the second is represented by T1E16-B that carries an extended 3'-end resulting from incorporation of an L1 sequence. Hence, all "XT" transcripts (Figure 1C) were complemented with the extended 3'-end of T1E16-B whilst the remaining *T1 *transcripts were 3'-tagged with the 3'-terminal sequence of the testicular *T1 *sequence (Figure 2A). For *T2*, all transcript sequences were tagged with the only 3'-end of the testicular *T2 *derived by 3'-RACE (Figure 2B). To discern possible exon organisation, the transcript sequences were used to query the rat genome sequence (assembly version RGSC v3.4, as on December 1, 2008). All discernible exon sequences were qualified by the presence of the consensus splice junctions. Furthermore, all transcript sequences were also subjected to scrutiny for repetitive sequences by querying the GIRI RepBase database. It is noted in the RepBase analysis that the ubiquitous exon 3 of *T1 *(T1–3) and exon 2 of *T2 *(T2-2) are derivatives of L1 and the endogenous viral (ERV) TE sequences, respectively. The derived exon organisation of the *T1 *and *T2 *transcripts is displayed in Figure 2. In the *T1 *transcript category, transcripts identical to the testicular *T1 *are detected at E15 (T1E15-RT2) and E16 (T1E16-RT2) but not at the E14-stage of development. Most developmental transcripts retain either or both the testicular exons 2 and 3 (T1–2 and -3) except for T1E14-XT2 that has lost both exons. Furthermore, T1E14-RT1 has retained the 534-bp intron separating exons 2 and 3. More significantly, seven *T1 *transcripts carry superfluous exons sandwiched between exons 1a and 2 (T1-2) (Figure 2A). The T1E16-B transcript appears to embody all these superfluous exons in two clusters derived from two distinct genomic sequences, designated as L1-ERV and S1-hAT for convenience of description, whereas other transcripts carry alternatively spliced segments of these two genomic sequences. The L1-ERV sequence was a composition of a ~170-bp relic sequence of the highly repetitive L1 fused to a short remnant of ERV sequence (Figure 3A; see also Additional file 1, panel A for sequence details). The S1-hAT genomic segment is a ~1.9-kb sequence composed of eight short interspersed element 1 (SINE1) remnant sequences and the hAT (for *hobo *from *Drosophila*, *Ac *from maize, and *Tam3 *from snapdragon) DNA transposon [26,27] (Figure 3B; also Additional file 1, panel B). In a BLAST query, the S1-hAT sequence was further found to be duplicated in tandem in chromosome 2 and an almost identical copy was also discerned in chromosome 3 of the rat genome (Figure 3B). Different spliced segments of these two genomic sequences are stitched to different *T1 *transcripts using the splice sites depicted in Figure 3. For example, the all-encompassing T1E16-B transcript harbours two L1 and two ERV subfragments of these two TE genomic sequences (Figures 2A and 3). Incorporation of extraneous TE-associated exons results in extended 5'-UTRs in the *T1 *transcripts with potential biological consequences as is demonstrated below.

On the other hand, the developmental *T2 *transcripts are uncomplicated in exon organisation (Figure 2B). All developmental *T2 *transcripts also carry the leader exon 1a and the uninterrupted coding exon 3 (exon T2-3). The testicular exon 2 (T2-2), which itself is an ERV remnant, may or may not be associated with the developmental *T2 *transcripts. In T2E16-RT1, a superfluous exon derived from L1 is found located between the constitutive exon 1a and the coding exon 3 replacing exon T2-2 (Figure 2B). The *T2 *transcripts may be simpler in exon organisation but they are still tinted by TE-derived exons.

In summary, sequence analysis of the *T1 *and *T2 *transcripts indicates extensive alternative splicing events involving sequences of various highly repetitive TE sequences contributing to the 5'-UTR of the transcripts, particularly the *T1 *transcripts. In the 3'-UTR, two major termination sites were elucidated for the *T1 *transcripts with the 3'-distal termination site contributed by L1. It is also observed that the overall exon organisation of the *T2 *transcripts is uncomplicated whereas the *T1 *transcripts vary extensively in the 5'-UTR structure when expressed during development.

### Detection of *T1-T2 *chimeric transcripts

**Figure 4:**
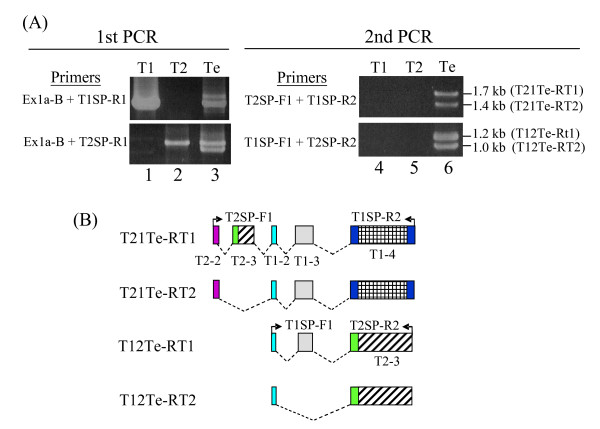
**Detection of *T1–T2 *chimeric transcripts in the testis**. **(A) **RT-PCR profiling of *T1–T2 *chimeric transcripts. Two rounds of nested PCR were performed using oligo(dT)-primed RT products of the testis mRNA. In the first-round PCR, the consensus Ex1a-B and the *T1*- or *T2*-specific 3'-UTR primers, T1SP-R1 and T2SP-R1, respectively, were used (see Figure 1A for relative positions and Table 2 for sequence details); in the second-round PCR, the exon 3 (*T1*) or exon 2 (*T2*)-based T2SP-F1 or T1SP-F1 primers were used in combination with T1SP-R1 and T2SP-R1. The *T1 *and *T2 *plasmids were used as controls in both rounds of PCR (lanes 1–2 and 4–5). The PCR products (with designations in brackets) generated in the second-round PCR (lane 6) were cloned and sequenced. **(B) **Schematic depiction of the PCR products derived from **(A)**. The exons of the transcripts are shown using the same colour code and exon designation as in Figure 2. Primers used in the second-round PCR are also shown.

In our previous work, we presented a testicular transcript which was a composite of *T1 *and *T2 *sequences [20]. To authenticate *T1-T2 *chimerism in this work, RT-PCR was performed using a *T1*- or *T2*-exon 2-specific forward primer in mix-gene combinations with a *T2 *or *T1 *reverse primers located at the 3'-UTR of the respective gene for detection of possible *T1-T2 *or *T2-T1 *exon constitutions. To increase sensitivity and specificity, two rounds of nested PCR were performed: the first-round PCR was done using an exon 1a primer (Ex1a-B in Figure 1A) common to both *T1 *and *T2*, and a *T1 *or *T2 *3'-UTR sequence-specific reverse primer (T1SP-R1 or T1SP-R2 in Figure 1A). In the PCR, plasmids carrying the *T1 *or *T2 *cDNA sequence were included as controls; these plasmids generated positive bands corresponding to the respective gene indicating gene specificity (Figure 4A, lanes 1 and 2). When the testicular mRNA was used in the first-round RT-PCR using the *T1 *or *T2 *primers, two major bands were discerned (Figure 4A, lane 3). For subsequent PCR, *T1- *or *T2*-specific exon 2 forward primer (T2SP-F1 or T1SP-F1) was used in a mix-gene fashion in combination with the *T2*- or *T1*-specific reverse primer (T1SP-R1 or T2SP-R2) located at the 3'-UTR sequence of the respective gene (Figure 4B; see also Figure 1A for primer map positions). In the control experiments in which the *T1 *and *T2 *plasmids were also tested in the mix-gene reactions, no PCR products were detected as expected for such mono-gene scenarios (Figure 4A, lanes 4 and 5). However, when the testicular first-round cDNA products were subjected to the mix-gene PCR, two distinct bands were now discerned in each of the mix-gene reactions (Figure 4A, lane 6).

The PCR bands were excised and cloned; two or more clones derived from each of these bands were sequenced; the sequences are given the prefixes T21 or T12 in the order of appearance of the *T1 *and *T2 *sequences. Exon mapping of the sequences derived clearly shows that the mix-gene PCR products are *T2–T1 *or *T1–T2 *chimeric transcripts defined by accurate splice-site demarcation (Figure 4B). The 1.7-kb T21Te-RT1 sequence is composed of the *T2 *exon 2 and partial exon 3 truncated within the coding sequence, and the *T2 *exons are accurately spliced to the *T1 *exons 2, 3 and the full coding sequence of exon 4. The 1.4-kb T21Te-RT2 sequence carries *T2 *exon 2 (T2-2) which is spliced to exons 2, 3 and 4 of *T1 *(T1–2, -3, and -4). Conversely, the 1.2-kb T12Te-RT1 sequence is composed of the *T1 *exon 2 (T1–2) spliced to exon 2 and the coding exon 3 of *T2 *(T2–3 and -3). The T12Te-RT2 sequence was simplest in structure in being composed of *T1*-exon 2 coupling with the coding *T2*-exon 3. Since these transcript sequences were RT-PCR products obtained rather forcefully through two rounds of PCR, they most likely represent minor populations of authentic *T1–T2 *chimeric transcripts. The naturally occurring 5'- and 3'-ends had not been determined for these chimeric transcripts. We have, thus, produced further experimental evidence that *T1–T2 *or *T2-T1 *chimeric transcripts do exist and their existence raises the question on how these transcripts are generated

### Embedment of *T1 *and *T2 *exons in a minefield of TE sequences

**Figure 5:**
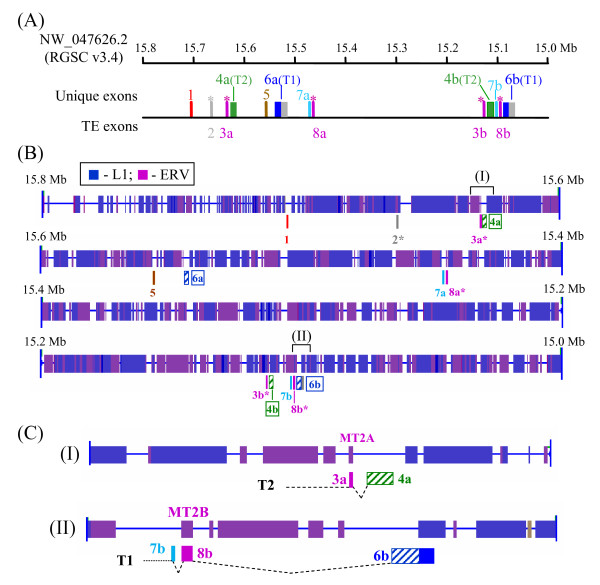
**Exon assemblage and exonisation of TE sequences into the 5'-UTRs of the *T1 *and *T2 *transcripts**. **(A) **A chromosomal map of discerned exons of *T1 *and *T2 *transcripts derived by bioinformatics-based alignment of the transcript and the genomic sequences. The relative map positions of the exons are in a linear order in the defined regional sequence of the Rn2_2148 supercontig. The new exon designations used are as defined in Table 1. Exons that are derived from TE sequences are asterisked with the exon designations shown below the exon map; exons of unique sequences are shown above the map. The *T1 *and *T2 *coding exons are shown in parenthesis. The scheme is prepared only to an approximate scale. **(B) **Embedment of *T1 *and *T2 *exons in a dense field of TE sequences. The nt 15,800,000–15,000,000 (15.8 Mb-15.0 Mb) chromosomal segment of the Rn2_2148 supercontig that harbours all the discerned *T1 *and *T2 *exons, the L1 (shown in blue) and the ERV sequences (in purple) are shown. The exons (see Table 1) are mapped below the TE sequences using the same colour code as in Figures 2 and 5A above. Coding exons are denoted by hatched bars and the boxed-in exon designations. Roofed segments (I) and (II) are shown in further details in **(C)**. **(C) **Expanded views of the roofed segments of the *T2 *and *T1 *aligning exons displayed in **(B) **above. The respective TE and coding sequences are shown.

Exon mapping reveals that the *T1 *and *T2 *exon sequences are dispersed over a ~700 kb segment in the sequence of the rat Rn2_2148 supercontig [GenBank:NW_047626.2]. Using a threshold of >98.5% sequence identity in BLAST-based queries, the relative physical locations of all the *T1 *and *T2 *exons are derived as shown in a linear order in Table 1 and Figure 5A. An important outcome of this analysis is the finding of duplicated cassettes of the *T2 *exons 2 and 3 (T2-2 and T2–3) each with a short intron of ~500 bp separating the exons (Table 1, solid boxes). For *T1*, there are also two cassettes of exons 2, 3 and 4 (T1–2, -3 and -4) (Table 1, dashed boxes). *T1 *exons 2–4 in the second (downstream) cassette are arranged in a linear order and are separated by relatively short introns. However, the exons in the first (upstream) cassette are arranged disorderly in a 4-2-3 exon configuration, and the coding exon 4 is mapped 64.6 kb upstream of the tight exons 2–3 doublet. A comprehensive exon map is constructed to include all the identified exons; the exons are also re-named based on the linearity of appearance and their presence in the *T1 *and *T2 *transcripts (Table 1 and Figure 5A). In the revised exon designation scheme, the numerical indicates the order of appearance of the exons; suffices "a" and "b" are used to denote different alleles of the same exons.

**Table 1:** *Rtdpoz-T1 *and -*T2 *exon assembly

Exon		Size (bp)	Rn_2148 position (nt)	Intron (kb)	Exon description
Previous	New				
1a	1	169	15,707,883 – 15,707,814	39.40	Major exon 1 for *T1 *and *T2*
L1-ERV	2		15,668,417 – 15,666,808	31.15	nonLTR-L1 & ERV TE sequences

T2-2	3a	157	15,635,659 – 15,635,503	0.563	ERV-MT2A TE sequence
*T2–3	*4a	**1,550**	**15,634,940 – 15,633,391**	**79.60**	***T2 *coding sequence**

S1hAT	5	279/36	15,553,789 – 15,550,897	9.98	Unique sequences amidst SINE/hAT

*T1–4	*6a	**1,530**	**15,540,915 – 15,539,386**	**64.6**	***T1 *coding sequence**
			**(15,538,842)**		**(3' L1 extension)**
T1–2	7a	73	15,474,800 – 15,474,727	0.534	Unique sequence
T1–3	8a	292	15,474,193 – 15,473,900	358.1	ERV-MT2B TE sequence

					

T2-2	3b	157	15,115,847 – 15,115,693	0.470	ERV-MT2A TE sequence
*T2–3	*4b	**1,550**	**15,115,223 – 15,113,674**	**12.45**	***T2 *coding sequence**

					

T1–2	7b	73	15,101,224 – 15,101,151	0.536	Unique
T1–3	8b	292	15,100,615 – 15,100,323	4.93	ERV-MT2B TE sequence
*T1–4	*6b	**2,075**	**15,095,393 – 15,093,863**	**--**	***T1 *coding sequence**
			**(15,093,321)**		**(3' L1 extension)**

Sequences with >98.5% identity between the *T1 *and *T2 *transcript sequences and the genomic sequence of the Rn2_2148 supercontig (NW_047626.2, RGSC v3.4) are listed. Exons are tabulated in order of appearance in the genomic sequences. Putative *T1 *and *T2 *coding exons are shown in heavy letters and are asterisked. The solid and dashed boxes denote the unique *T2*- and *T1*-specific exons, respectively, that are duplicated in different chromosomal locations. The relative exon positions are schematized in Fig. 5A.

As presented in the preceding sections, numerous *T1 *and *T2 *exons are derivatives of the highly repetitive sequences of the L1 and ERV transposable elements (Table 1 and Figures 2 and 3). When the ~700-kb genomic sequence that harbours all the *T1 *and *T2 *exons were subjected to a RepBase query, the sequence was found to be heavily mined with relics of TE sequences. An average TE occupancy of ~60% is computed; some segments contain as high as ~70% TE sequences (Figure 5B). In this genomic region, the unique *T1 *and *T2 *exons are precariously embedded within the TE minefield. When a transcription read-through primary transcript carries such a heavy loading of redundant TE sequences, TE sequences that have developed favourable splice junctions could readily be harvested as exons and be inducted into mature transcripts as typically exemplified by the *T1 *transcripts T1E16-A and T1E16-B (Figures 2 and 3). In the more simplistic *T2 *splicing, the ERV-MT2A has become an almost permanent landmark of the *T2 *transcripts harvested as exon 3a (T2-2) (Figure 5C, panel I). In the same token, the ERV-MT2B-derived exon 8a or 8b (T1-3) has also become a permanent fixture of the *T1 *transcripts (Figure 5C, panel II). At the 3'-end, transcription read-though of the regular transcription termination site into a downstream L1 sequence has also resulted in an extended 3'-UTR in a significant population of *T1 *transcripts (Figure 2A).

### A model of *T2 *and *T1 *transcription and post-transcriptional processing

Only one copy of exon 1 (previously called exon 1a) could be identified in the ~700-kb genomic sequence and also in the entire rat genome. When the completed mouse genome was interrogated for possible presence of the rat exon 1-like sequence, only one exon 1 copy was found on chromosome 3 [GenBank:NT_039240.7] with a 75.1% sequence identity but there were short ~50-bp segments showing >90% identities between the two sequences (see Additional File 2). When longer (1.5 kb) genomic sequences encompassing exon 1 and about 1-kb upstream sequences of the two rodents were aligned, sequence identity remained high at 69.4% (data not shown) indicating evolutionary relatedness. The described mouse-rat genomic sequence identity further supports the uniqueness of the exon 1 sequence in the rat genome. Interestingly, this exon 1 sequence is found only in the rodent genomes and not in the genomes of other animals and plants examined (data not shown) suggesting that the exon had evolved independently since the branching out of the rodent lineage.

**Figure 6:**
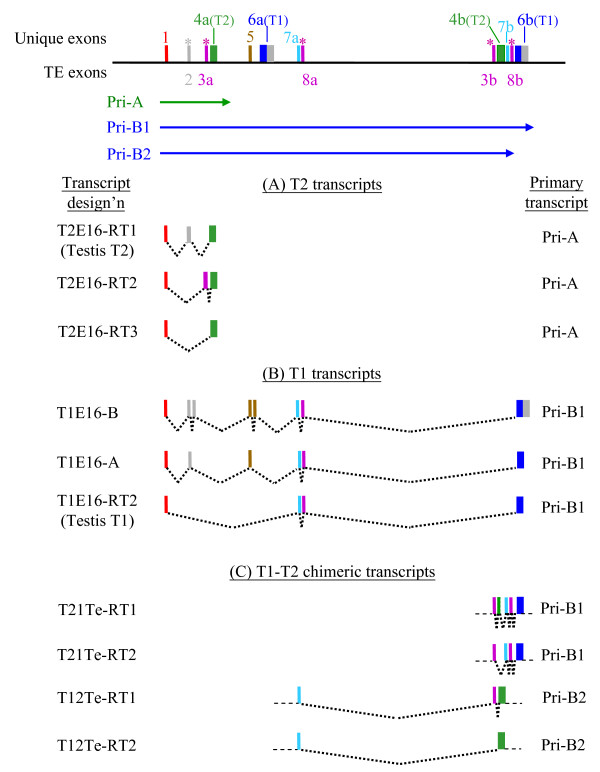
**A model of co-transcription, alternative splicing and exonization of TE sequences in the generation of the *T1 *(A), *T2 *(B) and *T1–T2 *chimeric (C) transcripts**. The ordered exon assemblage shown at the top of the scheme is as depicted in Figure 5A. Primary transcript A (*Pri-A*) is proposed for the generation of the *T2 *transcripts, and the transcription read-through primary transcripts *Pri-B1 *and *-B2 *are proposed for the generation of the *T1 *and *T1–T2 *chimeric transcripts, respectively, as explained in the text. In the figure, only transcripts that exemplify representative exon usages are shown. Exons 4a and 4b and exons 6a and 6b carry the *T2 *and *T1 *coding sequences, respectively, as indicated. Since chimeric sequences in panel **(C) **were derived by RT-PCR, only the derived exon sequences are shown. See also Figure 5 legend for an explanation of the symbols and colour code used.

Since all *T1 *and *T2 *transcripts carry the unique exon 1, our results clearly suggest that both genes are transcribed using a common promoter associated with this leader exon. Based on the derived exon order depicted in Figure 5A, the generation of the *T2 *transcripts is a simple and direct affair through *cis *splicing of the proposed *Pri-A *primary transcript (Figure 6, panel A). (Note that in Figure 6, the splicing of only representative transcripts is shown.) To explain the generation of the *T1 *transcripts initiating from the exon 1 promoter, transcription read-through of the *T2 *exon cluster and a run up to the 3'-distal exon cluster would have to be invoked to first generate a ~600-kb Pri-B1 primary transcript that terminates at the *T1*-coding exon 6b (or T1–4 in the previous terminology) followed by appropriate *cis *splicing (Figure 6, panel B). The downstream *T1*-coding exon 6b, but not the upstream exon 6a, would have been used due to the fact that the other preceding exons, exons 7a and 8a (T1–2 and -3), are in the correct linear order in the downstream cluster but not the upstream exon cluster (exons 6b-7a-8b). In this scheme, it could not, however, be ascertained if the upstream exons 7a-8a or the downstream 7b-8b exon doublet is used. To explain the generation of the *T1–T2 *chimeric transcripts, transcription initiating from the exon 1 promoter and the generation of the transcription read-through primary transcripts Pri-B1 and -B2 would have to occur followed by alternative splicing (Figure 6, panel C). For example, the T12Te-RT1 chimera would have used the upstream exon 7a (T1–2) that normally appears in *T1 *transcripts splicing to the downstream *T2 *noncoding exon 3b (T2-2) and the coding exon 4b (T2–3); the T21Te-RT1 chimera would have used the *T2 *exon 3b (T2-2) stitching to the *T1 *exons 7b-8b-6b cluster.

Taken together, the proposed exon organisation offers a model of co-transcription and post-transcriptional processing to explain the structure of the *T1 *and *T2 *transcripts. Firstly, the close proximity of the leader exon 1 with the *T2 *exons (exons 1, 3a and 4a) explains why the *T2 *transcripts are uncomplicated with fewer splice variants whereas the *T1 *transcripts are highly erratic and are frequently infused with TE sequences due to the extended size of the proposed primary transcripts. Secondly, the model also dissembles that chimeric transcripts in both the *T1–T2 *and *T2-T1 *orientations are generated as rogue transcripts that have acquired illegitimate exons of the cousin gene through erratic alternative splicing. Although there is no evidence of alternative *trans *splicing, this possibility could not, however, be completely ruled out.

### Translational repression by TE-derived uAUGs and uORFs of *T1 *transcripts

**Figure 7:**
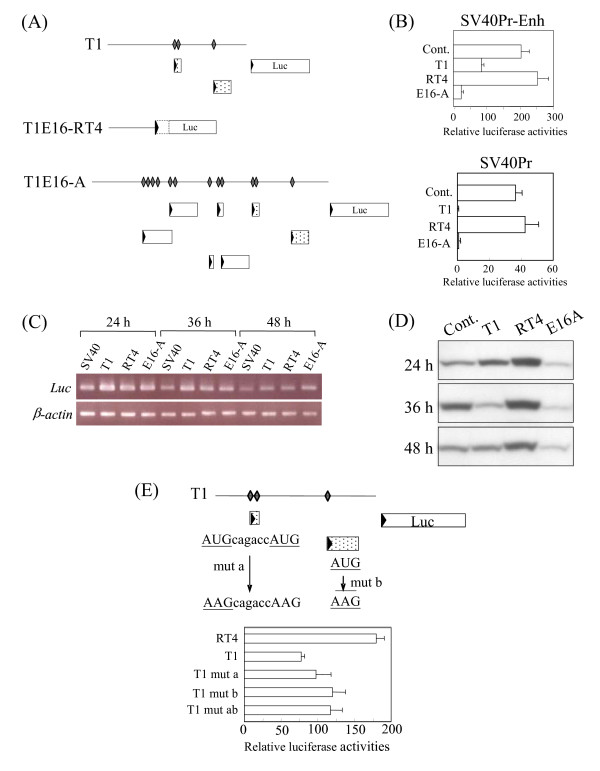
**Translational repression by uORFs of selected *T1 *transcripts**. **(A) **Identification of uAUG (depicted by diamonds) and uORFs (boxes, with the vertically positioned triangles indicating AUG) in the 5'-UTR sequences (horizontal lines) of selected *T1 *transcripts. Dotted boxes are uORFs found in the testicular *T1 *transcript. The depicted 5'-UTR sequences were placed before the luciferase (Luc) gene in the pGL3-Control (SV40Pr-Enh) or pGL3-Promoter (SV40Pr) vectors. In the T1E16-RT4 construct, a new initiation codon was fortuitously created by the cloning process adding 16 amino acid residues (dashed box) to the luciferase protein which did not affect the luciferase activity. **(B) **Transient transfection of CHO-K1 cells and luciferase assays using the constructs described in **(A)**. Transfection with the pGL3-Control or pGL2-Promoter plasmid was used as a positive control (Cont.). The data shown are from three independent experiments. **(C) **RT-PCR analysis of relative luciferase mRNA levels in the transfected CHO-K1 cells. Transfected cells were harvested at the indicated time points for total RNA preparation and semi-quantitative RT-PCR analysis using luciferase gene-specific primers (Table 2). **(D) **Western blot analysis of the luciferase protein levels in the transfected CHO-K1 cells at different post-transfection time points using an anti-luciferase antibody. The control (Cont.) data were derived from mock transfection using the blank vector. **(E) **Effects of disruption of uORFs on expression of the luciferase reporter gene. The three uAUGs and the two uORFs of the *T1 *transcript, shown in **(A) **above, were disrupted by site-specific mutagenesis at the initiation codons as shown: mut a was a double mutant of the two uAUGs (underlined) found in the first uORF; mut b removed the uAUG in the downstream uORF whereas mut ab was a mutant in both the up- and downstream uORFs. The mutagenised constructs were used in transient transfection of the CHO-K1 cells followed by luciferase assays. The data shown in the bottom panel were derived from three independent transfection experiments. The parental *T1 *and the uORF-free T1E16-RT4 were included for comparison.

In the 5'-UTR sequence of the testicular *T1 *transcript, we could discern three upstream AUGs (uAUGs) and two upstream open reading frames (uORFs) of 21- and 75-bp in length both of which are derived from the inserted TE sequence (exon 8b, or T1–3); the upstream 21-bp uORF is in the same reading frame with the *T1 *coding sequence (Figure 7A). The 5'-UTR-truncated T1E16-RT4 transcript lacks the TE insertions and, hence, the two uORFs in the 5'-UTR (Figure 7A). In the lengthy 5'-UTR of the T1E16-A transcript the bulk of which is composed of ERV and L1 sequences (Figure 2A), a total of twelve uAUGs and seven uORFs are discernible in all three reading frames with sizes ranging from 21 bp to 123 bp (Figure 7A).

To investigate if TE-containing 5'-UTR sequences of different *T1 *transcripts contribute to regulation of gene expression, the 5'-UTR sequences of the *T1*, T1E16-RT4 and T1E16-A transcripts were inserted before the luciferase gene under the regulation of the SV40 promoter and an SV40 enhancer; the constructs were used in transient transfection of the Chinese hamster ovary cell line, CHO-K1, followed by luciferase activity assays (Figure 7B). The results show that the luciferase activities under the regulation of the uAUG- and uORF-free T1E16-RT4 were maximal in the presence or absent of the SV40 enhancer. On the other hand, the uORF-abundant 5'-UTR of T1E16-A resulted in the lowest levels of luciferase activity whereas the *T1 *construct with two uORFs showed intermediate level of luciferase activity. Similar albeit lower relative luciferase activities were obtained using the testicular cancer cell line LC-540 in similar transfection and luciferase assays (data not shown).

To determine if the varied luciferase activities observed are associated with differential RNA stabilities, total RNA was prepared from the transfected cells at different post-transfection time points for RT-PCR analysis. At each of the time points examined, the luciferase mRNA level was found to be comparable for the three 5'-UTR constructs indicating similar luciferase mRNA stability despite the presence of different 5'-UTR sequences (Figure 7C). To discern possible regulation at the translation step, western blot analysis of lysate of the same sets of transfected cells was performed using an anti-luciferase antibody. The results show that the luciferase protein level was maximal for T1E16-RT4, minimal for T1E16-A and intermediate for *T1*, in direct agreement with the relative luciferase activities determined above (Figure 7D). The effects of uAUGs and uORFs on translation were further supported by data derived from mutagenesis analysis of the two uORFs of *T1 *in the luciferase constructs (Figure 7E). On removal of either or both the uORFs of *T1*, luciferase activities were partially restored. Our data collectively indicate that different 5'-UTR sequences in the *T1 *transcript variants carrying different numbers of TE-derived uAUGs and uORFs could result in repressed translation of the *T1 *gene.

## Discussion

In a previous work, we first described testis-specific transcription of the *Rtdpoz-T1 *and *-T2 *genes [20]. In this work, we show that *T1 *and *T2 *are also transcribed in the developing embryo (Figure 1). More significantly, we show that each of the uninterrupted *T1 *and *T2 *coding exons are duplicated and the exons are embedded in a dense field of TE sequences. Consequently, the embryonic *T1*/*T2 *transcription displays two novel features: co-transcription of the two genes and frequent exonisation of TE sequences into the 5'-UTRs of the transcripts.

### Developmentally-regulated *T2 *and *T1 *co-transcription

Co-transcription of *T2 *and *T1*, in this gene order, is proposed based on the observation that all testicular and developmental *T2 *and *T1 *transcripts discerned share a unique exon 1 that resides upstream of the *T2/T1 *exon assemblage (Figures 2 and 5A). The exon 1 sequence is also found to be conserved as a unique sequence in the mouse genome (see Additional file 2) but not in the genomes of other animals and plants examined, consistent with evolutionary relatedness between the mouse and rat genomes. *T2*-*T1 *co-transcription implies that the genes are co-ordinately controlled by a common promoter and associated regulatory sequences.

*T2 *transcription is found to occur throughout the E12 to E17 developmental stages analysed and is silenced beyond E17. On the other hand, *T1 *transcription is restricted only to E14–E16. In rodents, the organogenesis phase of embryonic development comes to an end at about E14–E15 from which point on active foetal growth occurs, a process that involves active cellular proliferation as opposed to active differentiation during the organogenesis phase [28]. Our data collectively suggest that *T2 *expression is a normal monogenic transcriptional event that uses the AAUAAA polyadenylation signal located 10 nucleotides upstream of the polyA tract of the mature *T2 *transcripts [GenBank:AY902367 and ref. [20]] and. On the other hand, *T1 *expression is only realised when *T2*-to-*T1 *transcription read-through occurs to transcribe the *T1 *coding exons (Figure 6). The occurrence of *T2*-to-*T1 *co-transcription may be attributed to high rates of transcription associated with active foetal growth similar to hyper transcription rates that have been shown in the testis [10]. The silencing of *T1/T2 *transcription at E17–E18 does not seem to involve hypermethylation of the exon 1 promoter which is unmethylated, despite the presence of a CpG island, in the testis and in all the developmental stages examined irrespective of *T1/T2 *expression (unpublished data). It remains to be investigated if developmental and testicular transcription of the *T2*/*T1 *locus involves chromatin remodelling or the availability of positive or negative *trans*-acting factors.

### Exonisation of TE sequences into 5'-UTRs of the *T1 *and *T2 *transcripts

The most notable finding of this work is the high rate of exonisation of TE sequences into the 5'-UTRs of the *T1 *and *T2 *transcripts through alternative splicing. In some *T1 *transcripts, an alternative transcription termination site is found in an L1 sequence located downstream of the constitutive site (Figure 2A). Frequent TE exonisation is clearly associated with the embedment of the constitutive exon 1 and the uninterrupted *T1 *and *T2 *coding exons in a ~700-kb chromosomal segment that is heavily populated with TE sequences (Figure 5). In this segment, the computed average TE content is 60.7%, much higher than the mean TE content of 40% in the rat genome [29,30]. Notably, the second exon of the *T2 *gene (T2-2 or exon 3a/3b) and that of *T1 *(T1–2 or exon 8a/8b) are relics of the ERV-MT2A and -MT2B sequences, respectively; these TE relics have developed strong and stable splice sites to be frequently recruited into 5'-UTRs of the transcripts of the respective gene (Table 1 and Figure 5). We also detected apparent *T2*-*T1 *"chimeric" transcripts that involve only 5'-UTRs (Figure 4). On closer examination, all the discerned *T2 *and *T1 *5'-UTR exons, with the exception of the constitutive exon 1 and the exon 2 of *T1 *(T1–2, or exon 7a/7b), are TE remnants (Figures 2A and 2B, see bottom TE annotations). However, we cannot rule out the possibility that the T1–2 exon was also originally derived from a TE sequence but had lost its TE features through evolution for recognition. In other words, all 5'-UTR exons, except exon 1, may, in fact, be products of exonised TE sequences recruited through alternative splicing.

Several salient features of TE insertions in the human and mouse genomes have been described based on bioinformatics analysis: (i) the TE exons are mostly intronic; (ii) all TE families can be exonised; (iii) TE exons are found mostly in the UTR, and (iv) potential tissue-specific association [31,32]. In this report, the depicted exon organization of the *T1 *and *T2 *transcripts has provided direct experimental evidence to support all of the above features of TE-derived exons. An important mechanism that contributes to the exonisation of Alu, a highly repetitive and primate-specific TE, is the RNA-editing-mediated adenosine-to-inosine (A-to-I) modification [33-35]. A-to-I RNA editing is catalysed by adenosine deaminase acting on double-stranded RNA stretches of primary transcripts formed by annealing of inverted-repeat sequences in the pre-mRNA [34]. The dense field of predominantly LINE1 and ERV sequences in the *T2/T1 *locus provides ample opportunities for the *T2/T1 *pre-mRNA species to form double-stranded structures for adenosine deaminase-mediated RNA editing. Furthermore, the TE exonization may be driven by the use of cryptic exonic splicing enhancers (ESEs) as proposed by Lin et al. [32]. The exact mechanism that is responsible for exonisation of TE sequences into the *T1 *and *T2 *transcripts is a subject for further investigation.

Biologically, TE insertions into 5'-UTRs of transcripts have been shown to influence gene expression at the level of transcription through the creation of new transcription factor binding sites or by other transcriptional mechanisms [36-38]. Alternatively, the presence of TE sequences could introduce deleterious uAUGs and uORFs to repress translational initiation [39-41] as we have demonstrated for selected *T1 *transcripts (Figure 7). The complexity of the *T1 *transcript population in the developing embryo impedes detailed determination of the relative abundances of the discerned transcripts.

## Conclusion

This work provides evidence to indicate that exonisation of TE sequences is a frequent event in the transcription of retrogenes during embryonic development and in the testis and TE exonisation may contribute to post-transcriptional regulation of expression of retrogenes through translational repression. The *T2/T1 *locus, thus, provide a spatio-temporal model for further dissection of developmentally-regulated and testis-specific transcription and possible biological significance of TE exonisation of retrogenes.

## Methods

### Cell lines and rats

The rat insulinoma cell line RIN-m5F was acquired from the Bioresource Collection and Research Centre, Taiwan. Sprague Dawley rats were used throughout this work and were obtained from the Laboratory Animal Centre, National Yang-Ming University, Taiwan. This study was approved by the Institutional Animal Care and Use Committee (IACUC) of the Taipei Veterans General Hospital. The animals were sacrificed according to the IACUC guidelines.

### RNA preparation and expression profiling by RT-PCR

Total RNAs were prepared from rat tissues using the TRI-Reagent^® ^(MRC, Cincinnati, OH) and were treated with DNase before reverse transcription. Total RNA from whole embryos from the embryonic stages E12 to E20 were purchased from Zyagen. RT-PCR-based expression profiling was performed as described [15,20]. Briefly, five microgram aliquots of total RNA were initially used to generate the first-strand cDNA using an oligo(dT) primer and the SuperScript^® ^II First-Strand Synthesis System (Invtirogen). For the standard expression profiling (Figure 1B), the RT products were subjected to a first-round PCR using the Ex1a-B + T1SP-R1 or Ex1a-B + T2SP-R1 primer pairs (see Table 2 for primer sequences and the relative positions as depicted in Figure 1A) and the following PCR conditions using the Fast-Run^® ^Taq Master Mix Kit (Protech): 94°C for 3 min for initial denaturation followed by 35 cycles at 94°C for 30 sec, 59°C (for *T1*) or 61°C (for *T2*) for 30 sec, 72°C for 3 min and the reaction was further extended at 72°C for 10 min before termination of the reaction. The first-round PCR products were diluted 200-fold before being used in the second-round PCR using the Ex1a-A + T1SP-R3 or Ex1a-A + T2SP-R3 primer pairs (Table 2 and Figure 1A) as described above except for 25 cycles of amplification and with an annealing temperature of 57°C or 51°C for *T1 *or *T2*, respectively. The *T1 *extended PCR analysis (Figure 1C) was performed essentially as for the standard profiling PCR as follows: For the first-round PCR, undiluted RT product was subjected to amplification using the primers Ex1a-B and 3096R (Table 2 and Figure 1A) and an annealing temperature of 61°C for 35 cycles. The first-round PCR products were diluted 100 times for the second-round PCR using the primers Ex1a-A and 2965R (Table 2 and Figure 1A). The PCR conditions are as in the first-round PCR except that the primer annealing temperature was at 68°C. In all expression profiling experiments, β-actin gene-specific primers were included as an internal control in all the RT-PCR experiments. Specific discrimination between *T1 *and *T2 *in the expression profiling was demonstrated by the use of *T1 *or *T2 *sequence-containing plasmid DNA originally derived from the testis in the previous study [20]; these plasmid controls resulted in positive bands only in the designated reactions (data not shown but a demonstration of a similar assay is shown in Figure 3A).

**Table 2:** Oligonucleotide primers used in this study

Primers	Sequence (5'-3')
	(A) Primers for RT-PCR expression profiling
Ex1a-B	CAGGGAGACAGCTGATACATTTAG
Ex1a-A	TCCAGCCAGAGAAAGACTCATCATC
T1SP-F1	CCCACTTCTTCAGAATTCCAGCAT
T1SP-R1	GTTCAAGACATCAGACAAGAATAA
T1SP-R2	CTTTAGTCTTCAGGTTCTCTGCTC
T1SP-R3	AACATCTGAGATCTTTGTAATAAC
T2SP-F1	CGCACATCTTCAGGATTCTGGTCG
T2SP-R1	AGACATCAAACAAGAATCCAACAG
T2SP-R2	CCCTAGTCTTCAGGTGCTCTGTTT
T2SP-R3	AACATTTGACATTTTTGTAATAAG
2965R	GAGATTGCCACACAGATGATAGAG
3096R	TTGACCATATAAACCAACTGACAG
	(B) 5'- and 3'-RACE gene-specific primers
5' RACE-GSP-R1	CACCAGGCTGCAGTCTGTGAAGAGG
5' RACE-GSP-R2	CTTCACTGGCCTCTAATGAGAACC
3' RACE-GSP-F1	AGATTACCTGTCTGTTTACCTGGG
3' RACE-GSP-F2	TTGGGCAAAGGTACAGTTCTGGAT
	(C) Primers for cloning of 5'-UTR for uORF assays
T1 uORF-F	TTGCGTCCGGCTCTCAGC
T1 uORF-R NcoI	AACCATGGTTTCTGGAGGTAGTTTGGA
	(D) Primers for site-directed mutagenesis
F215	CCTGAAGAGTAAGCAGACCAAGAGAACATAGAG
R247	CTCTATGTTCTCTTGGTCTGCTTACTCTTCAGG
F373	GTCTATATAAGCTCAAGGAAGAAGGAAGC
R401	GCTTCCTTCTTCCTTGAGCTTATATAGAC
	(E) Primers for luciferase RT-PCR
Luc-F (1265–1288)	ACATAGCTTACTGGACGAAGACG
Luc-R (1557–1580)	GTAAGACCTTTCGGTACTTCGTCC

### 5'- and 3'-RACE and bioinformatics analysis

The procedure of rapid amplification of cDNA ends (RACE) was used to derive sequences of the 5'- and 3'-halves of *T1 *and *T2 *mRNAs for construction of full-length transcript sequences. For the RACE experiments, a SMART^® ^RACE cDNA Amplification Kit (BD Biosciences) was used according to the manufacturer's instructions and as described [1,20]. To increase specificity and sensitivity, nested PCR was routinely performed using the Nested Universal Primer A included in the SMART kit and *T1- *or *T2*-gene specific nested primers (5'RACE-GSP-R2 or 3'RACE-GSP-F1 for 5'- or 3'-RACE, respectively, see Table 2). All RACE-generated sequences were cloned into the pGEM^®^-TEasy vector for sequence analysis. Nucleotide sequences were subjected to BLAST searches of the GenBank rat resources database [; assembly version RGSC v3.4, as on December 1, 2008] using default parameters and filters. The Lasergene^® ^software programs package obtained from DNAstar^® ^was used for in-house sequence alignment and nucleotide sequence analysis.

### Plasmid construction, site-specific mutagenesis, transient transfection and luciferase activity assay

The 5'-UTR sequences of selected *T1 *transcripts were derived from the RNA by PCR amplification using oligo(dT)-primed RT products and reverse primers flanked with NcoI recognition sequence for cloning into the SV40 promoter-driven pGL3-Promoter and the SV40 promoter-plus-enhancer pGL3-Control luciferase reporter plasmids (Promega). For site-directed mutagenesis, oligonucleotides encompassing the mutations and containing restriction cloning sites were used as primers in PCR amplification reactions as described [42]. For transient transfection experiments, CHO-K1 cells were seeded onto 24-well Petri dishes 24 h prior to transfection. Cells were co-transfected in duplicates with the luciferase constructs and the thymidine kinase promoter-driven *Renilla *Luciferase plasmid using the Lipofectamine Plus reagent (Life Technologies) as described [20,43]. Forty-eight hours post-transfection, cells were lysed and duplicates of 20 μl aliquots of the cell lysate were removed for measurement of the luciferase activities in a luminometer using the Dual-Luciferase Reporter Assay Kit according to the user's manual (Promega).

### Western blot analysis

CHO-K1 cells transfected with *Luciferase *reporter constructs were harvested 24, 36 and 48 h after transfection. Subsequent processing of the lysed cells for western blot analysis using an anti-luciferase antibody (Novus) was performed as previously described [42]. Signals were visualised by chemiluminescence after treating the membrane blot with a Western Lightning Plus-ECL reagent (Perkin-Elmer) according to the manufacturer's recommendations.

## Authors' contributions

CJH and KBC conceived and designed all the experiments and did the data analysis in the study; WYL and CMC performed all the experiments; CJH and KBC wrote the paper.

## Supplementary Material

Additional file 1 **Splice sites in the TE-associated genomic segments that contribute to the 5'-UTR of the *T1 *transcripts**. The splice sites in the (A) L1/ERV and (B) S1-hAT TE sequences are as defined in the text. In the sequences, exons and introns are shown in upper- and lowercase letters, respectively. The 5'- and 3'-splice sites (5'- and 3'-ss) are shown. At the bottom of each sequence, the GIRI RepBase-derived tabulation of the TE sequences is also shown. Click here for file

Additional file 2 **Alignment of the rat (Rn) and mouse (Mm) exon 1 sequence**. The Mm and Rn exon 1 sequences are the mouse and rat genomic sequences that align with the common leader exon 1 of the rat *T1 *and *T2 *transcripts. The overall identity between the mouse and rat exon 1 genomic sequences is determined to be 75.1%. The figure is taken from an NCBI BLAST alignment. Click here for file
