# Supplementary material for: Transcription of the rat testis-specific Rtdpoz-T1 and -T2 retrogenes during embryo development: co-transcription and frequent exonisation of transposable element sequences
Source: BMC Mol Biol. 2009 Jul 25;10:74. doi: 10.1186/1471-2199-10-74 (PMC2724483; doi:10.1186/1471-2199-10-74)

**Additional file 1**

**Panel (A). Splice sites in the L1/ERV TE sequences.**

**(L1)**gggatagtgattcccccagaagtccttttattgttgaggatagttttagctatcctgggttttttttttattcc***ag***

3’ss-1

ATGAATTTGCAAATTGTTCTGTCGAACTCTCTGAAGAATTGGATTGGTATTTTGATGGGGATTGCATTGAACCTGATGCC

CTTCAACAGTGGAATGGATACAGAAAAT***GT***GGTACATCTACACAATGGAATACTACTCAGCTATCAAAAACAATGACTTC

5’ss-1

ATGGAATGCATAGGAAAGTGGAATGAACTAGAAAATATCATCCTGAGTGAG***GT***AACCCAGTCAGAGAAAAACACACTTGG

5’ss-2

TATGCACTCATTGATAAGTGGATATTAGCCAAAAGGCTGGAATTACCCAAGATGCAATCCACAGATCACAGGAAGCTCAA

GAAGAAGGATGACCAAAATGCAG***GT***GCTCCCACTCCTTTTTTTTTTATTAACTTGAGTATTTCTTATTTACACTTCGAGT

5’ss-3

GTTATTCCCTTTCCTGGTTTCCGGGCAAACATCCCCCTAATCCCTCCCTCTCCCCTTCTTTATGGGTGTTCCCCTCCCCA

TCCTCCCCCCATTGCCGCCCTCCCTCCAACAATCACGTTCACTGGGGGTTCAGTCTTAGCAGGACCAAGGGCTTCCCCTT

CCACTGGTGCTCTTACTAGGATATTCATTGCTACCTATGAGGTCAGAGTCCAGGGTCAGTCCATGTATAGTCTTTAGGTA

GGCTCCCACTCCTTAAAAGTGGAAAAATATCCATAGTAGTGGTTATGGAAGCAAAGTTTAGAGCAGTGACTGAAGGAATG

GCCATTCAGAGCCTGCCCCACATGTGACCCATATATATACAACCACCAAAGCAAGATAAGATTGATGTAGCTAAAAAAAT

GCTTGCTGAAAGGGACTGGATATAGATCTCTCTTGAGAGACACATCCAGATCATGTTCAATACAGAGGTTAACACTTGTT

GCAAACCATTGAACTGAGAACAGGACCCCCTTGGGGGGAATTATAGGAAGGATTGAAAGAGTTGAAGGAGCTTGCAACCC

CATCAGAACAATACTGCCAACCAACTAGAGCTTCCAGGGACTAAACCACTACCGGAAGACTACACAAGGCCTGACCCAGG

GCTCCAACTGCATATGTAGCAGAGAATAGCCTTGTTGGGGCACCAGTGGAAGGGGAAGCCCTTGGCCCTGCCAAGGTTGG

ACCCCCACTGCAGGGGAATGTGGGGGGGTGTTAGGGGAGTCATTTTGTGGGAACACCCATATGGGTGAGGGGGATAGGAT

GGCGGGGGGCTTATGGACAGGAAACCGGGAAAGGGAATAACATTTGAAATGTAAATAAATAAACATATCTAATTATAAAA

//**(ERV)**AGAGTCACAGGGAGGGACAGGAGACCTGGAGCACAACTGCTTTCAAGGAATTTTTCCAGCAGACATACGGAA

TAGGTCTGGACATCTAAACCCTGGATAGCCATTTTGTGTTTACTTCCCCGTATCCCCCCACATCTACAC***AG***GAAATCGAC

3’ss-2

AGACAGACTGGATCTTTGGAGTGTTTGCTTGAGCTTCAGGTAGCCTAAGATCAACACAGAAGCAAGCCCTGCCAACTGCA

AGGGAAAGAGGCCCCTTGCTGTGAAAAAATAGGAGAGGACTGCAGTGTTCTGAATCGAACAGCAGAGGGAGACAAAGGAA

CAAAGGGCTCCAGATCTG***gt***aatacttgatataaacagagatatataatcttttgatacttaaggatgagaaacacaata 5’ss-4


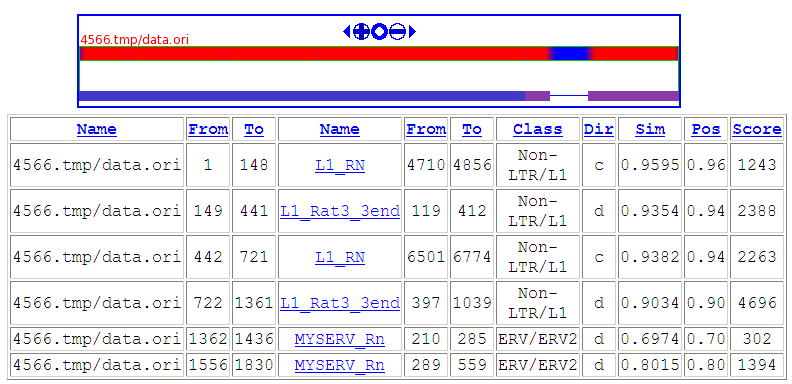


**Additional file 1**

**Panel (B). Splice sites in the S1-hAT sequence.**

nt 15,554,256

tgttccagggtagctagagttacaagaaaacaggcagaagctgggtatggtggtacatggctgcaatcctaggacttcag

aggctgagacagatgaattggcaaacttgagaccaatctggactacatagcaagattattaaagaaagaggatgaagaag

aagagggagggaggaaggaaacagcacagcaccggcttcctgaatcagtgttagtgaaagacctcactatctcctaccat

gaggaaaaactgctgagggactgaaagaatgactggatttattcattcctgggcttattcagaaatacaagacactggga

cttgtagagaaatgggaatgcatggtgtacagtattcgcatagatgtaagctcagaatacacacaggactctaacacagc

tcagagggcactcgtaaagaagagggctgaacttgtagaatatttgttcaatctctgcttctagc***ag***GTTCTGCAGGGAT

**3’ss-1**

AACATGCTTCTGTGTCAGAAGTTAAAGAGATACCTAGGAAGTGGCTCTGTCTTCAACATCCCTTTACTCCTGGGCGAGAT

TGCTAGACGCCTTCTTTGGTGAGAAAAAGCTTCAGAGAAGAGAAGACTTGGAAAGCAATTCTAAAGAAATGCCCGGAAGC

AGTACCTGAGACTTGACCACCCAATGGGATCCAGACAAGATTATGGTTGAGGAGAGCCAGAGCACCCCAAGAAAAAGAAA

AAGGAACAGCATAGAAGCTGAGAG***GT***AGGGATGTGGTGATAGGTACTCTTTATTGTTTGGGGAGGCCTCAAAACCTCTGA

**5’ss-1**

GAAAAAACAGGAAGAGTGGAAGTTACTGAGAAAAACAGGAAGAGTGGAAAAGCAAGCTAGGGCAGACTCTAGAGTTCCTG

TAGCCACTGCTCAGCTGCAAAGACATCATTTTTCTTGAAGGTTCCCTCCCCTTTCCCTTACTGCCTTCGTCTAGCTGGTC

TATCCTTAGCTCTCGGATGTGTTTGCCTTGGTCTGAAAGTCCTCTGTTCACCAAGACTTCTCCCTGCAGACAGAGCAGAC

TCTCCTTCCCCATTAGAGGACTGATTCTCTTGGCTCTGCCCATGTATAATCATATGTGTGAAGTGTTTCCTTTTCCATTC

AC***AG***CCTTCCAAAGCCCTGCTTGACCCCATCTCTTCTCAG***gt***aagagagcagaacagcttctcaggaaagagcagactca

**3’ss-2**  **5’ss-2**

ctgaagcaggttgtcagtaagagcctcttcagtttcccctcacccctcagtaaggtggctttttaatcttcactgtctgg

cttctctcctgtgctttcacgtctggcaaccgacagtatggaagactctggatggcctcaaacacgttgttggctactag

gtgcgcaaagcaaagaagtacacagaaggcacatcaaaagggacaggttcaaacagacatgagactctttgttctgaagt

gcattcctttctctgaggtcttcatgctaaccttgcttcagcaattgccctaatgactatctccgatgcctttccctaag

ctaggggaaaggtcagcaaggaacgttcagaaacctactcctgcatctgcctatctttgtgtacataaagctcaccagaa

cacaactgtgctccttaatgaatatactccctttgctattttctgcaaccaagctgagtgattgcagctgaaaacatggc

tggcaaaagcctggcatgtttactacctagcgctttacaataaaaagcttgccaaatgccttgcttatgagaagttttag

actttgttcattttagaaaaatgtaagatgggcaaccgagaaggacctggaaaggtggctgggacatcatcccagcaccc

cgaagtcgaggcaggagtactgtttgagtttgaagccagccaggactacataatgagttacaggctagcctgagctacac

catgataagaccctgtctgaaacaacaaacacgaaagagaatgaaaaaagggatagaatgggagaagagcaaacggaaaa

gaggaaaagggagtacaagtaggcaaccgagatgcctaacaatgacgtagggagggacacaatccactctgcatctcagc

ctccgtggaccaatccatgtcttctctcagtctgttccaaaacactgagcagtactagctccactctagctcttcttttc

attcttctgttctgttcttttatttcaaattaatgcataacaatcacaagtttacaatgtatcaacaatccagtcaggat

aatgaacaggtacctctccttttttttctagtttttctttcagaagttatacaaattgctacgacttttcctcttgggta

agtgactcagtgtccatcactcatatatcctctgttttctctgcacnnnnnnnnnnnnnnnnnnnnnnnnnnnnnnnnnn

nnnnncagnnnnnnnagcnnnnnnnnnnnnnnnnnagacnnnanttttcagtttcnacatatgnnnnnnncacgcatcca

gagaacgtacagcanctgtttcagtagttctnncatgattcatttaccgaccnanttnnaagttcttttttaaatttgtg

tatgtggggagtggataagaaaacatgggtgtgagtgtactaggaaaccagaggcatcatatctccctgcagctgaaatt

acaagtggatgtaagctaccagatgtgaatgttgggaactgaatgcaggtcttctgcaagagcagtgttcacttttaagc

actgagccatccatctctccagctctactcactcacaaaaattattatcatacttttaaagccaggtgtggtggtacttg

tctttaattccagcccttgggaggcagatctctgagagctcaaggccagcctggcctacagagcgagttccaggacagcc

agaaatatagagagaccttgtctcaattttttttattatatgtgattatttatgtgtatctcacgtggaggtcagaggac

aaaatttggaagctctttcctttcttgtgggtctgaggaattgaactcaggtctccaggcttggtagccagcatctcgac

cgnnnnngccatttcaccagccatacgtgabxandnsnnnnnnnnnnnnnnnnnnnnnnaannnnnnnnnnnnnnnnnnn

nnnccttagctctcggatgtgtttgccttggtctgaaagtcctctgttcaccaagacttctccctgcagacagagcagac

tctccttccccattagaggactgattctcttggctctgcccatgtataatcatatgtgtgaagtgtttccttttccattc

acagccttccaaagccctgcttgaccccatctcttctcaggtaagagagcagaacagcttctcaggaaagagcagactca

ctgaagcaggttgtcagtaagagcctcttcagtttcccctcacccctcagtaaggtggctttttatcttcactgtctggc

ttctctcctgtgctttcacgtctggcaactgacagtatggaagactctggatggcctcaaacacgttgttggctactagg

tgcgcaaagcaaagaagtacacagaaggcacatcaaaagggacaggttcaaacagacatgagactctttgttctgaagtg

cattcctttctctgaggtcttcatgctaaccttgcttcagcaattgccctaatgactatctccgatgcctttccctaagc

taggggaaaggtcagcaaggaacgttcagaaacctactcctgcatctgcctatctttgtgtacataaagctcaccagaac

acaactgtgctccttaatgaatatactccctttgctattttctgcaaccaagctgagtgattgcagctgaaaacatggct

ggcaaaagcctggcatgtttactacctagtgctttacaataaaaagcttgccaaatgcctcgcttatgagaagttttaga

ctttgttcattttagaaaaatgtaagatgggcaaccgagaaggacctggaaaggtggctgggacatcatcccagcacccc

gaagtcgaggcaggagtactgtttgagtttgaagccagccaggactacataatgagttacaggctagcctgagctacacc

atgataagaccctgtctgaaacaacaaacacgaaagagaatgaaaaaagggatagaatgggagaagagcaaacggaaaag

aggaaaagggagtacaagtaggcaaccgagatgcctaacaatgacgtagggagggacacagtccactctgcatctcagcc

tccgtggaccaatccatgtcttctctcagtctgttccaaaacactgagcagtactagctccactctagctcttcttttca

ttcttctgttctgttctttt nt 15,549,875


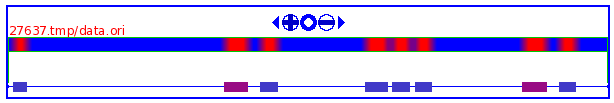

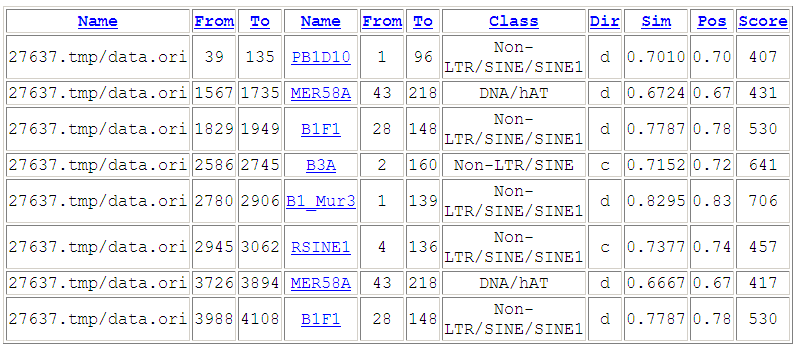

Supplement: Additional file 1 — Splice sites in the TE-associated genomic segments that contribute to the 5'-UTR of the T1 transcripts. The splice sites in the (A) L1/ERV and (B) S1-hAT TE sequences are as defined in the text. In the sequences, exons and introns are shown in upper- and lowercase letters, respectively. The 5'- and 3'-splice sites (5'- and 3'-ss) are shown. At the bottom of each sequence, the GIRI RepBase-derived tabulation of the TE sequences is also shown. [file 1471-2199-10-74-S1.doc]
