# Supplementary figures and images for: Transcription of the rat testis-specific Rtdpoz-T1 and -T2 retrogenes during embryo development: co-transcription and frequent exonisation of transposable element sequences
Source: BMC Mol Biol. 2009 Jul 25;10:74. doi: 10.1186/1471-2199-10-74 (PMC2724483; doi:10.1186/1471-2199-10-74)

## Slide 1
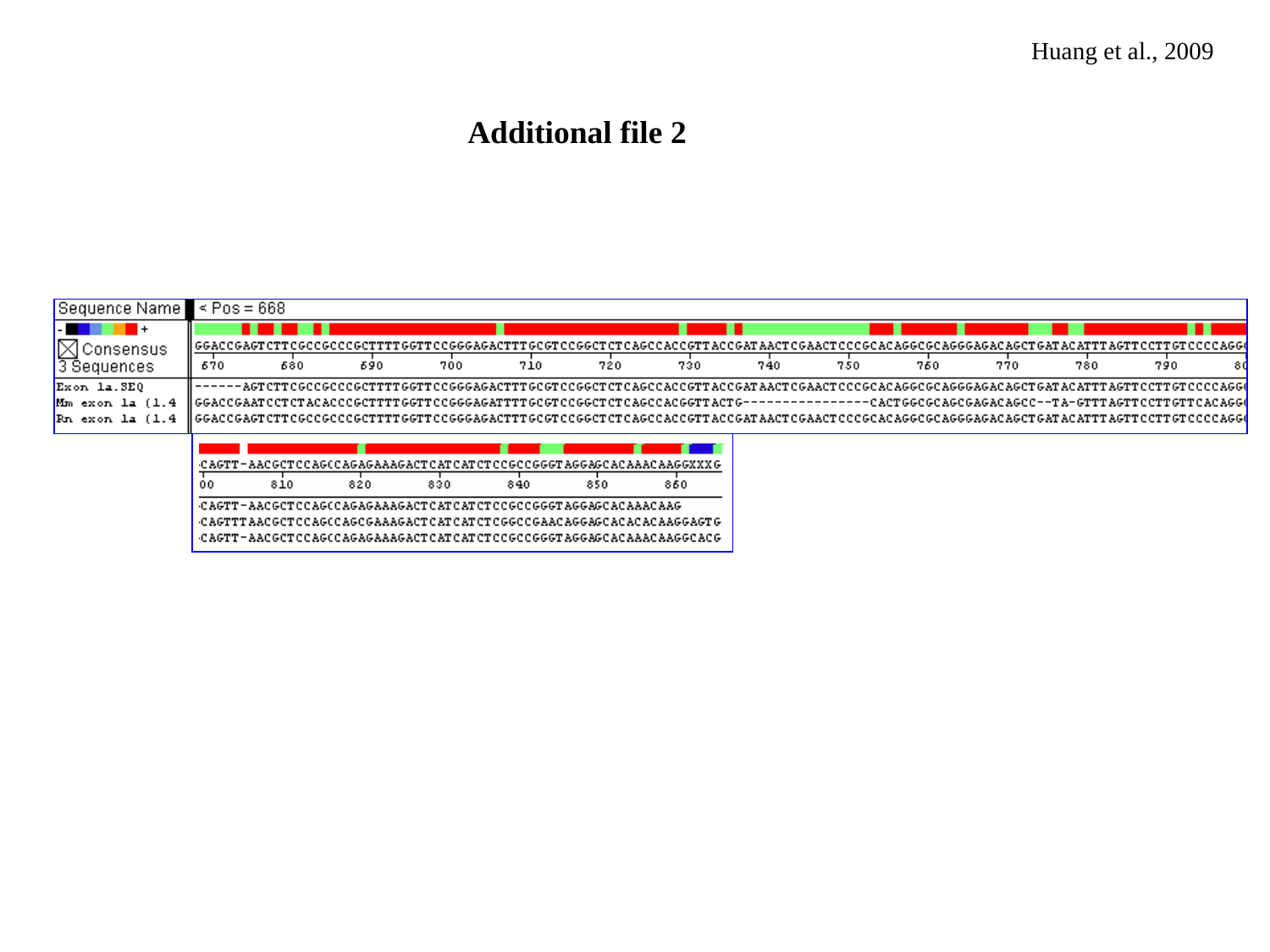

Huang et al., 2009
Additional file 2

Supplement: Additional file 2 — Alignment of the rat (Rn) and mouse (Mm) exon 1 sequence. The Mm and Rn exon 1 sequences are the mouse and rat genomic sequences that align with the common leader exon 1 of the rat T1 and T2 transcripts. The overall identity between the mouse and rat exon 1 genomic sequences is determined to be 75.1%. The figure is taken from an NCBI BLAST alignment. [file 1471-2199-10-74-S2.ppt]
